# Supplementary material for: Yeast encapsulation of photosensitive insecticides increases toxicity against mosquito larvae while protecting microorganisms
Source: PLoS One. 2024 Oct 29;19(10):e0310177. doi: 10.1371/journal.pone.0310177 (PMC11521277; doi:10.1371/journal.pone.0310177)
Supplement: S1 Fig — Larvae were incubated in the dark for 2 hr in water and either 0.002% (10 μL), 0.004% (20 μL), 0.01% (50 μL), 0.02% (100 μL), or 0.04% (200 μL) ethanol, for a total volume of 5 mL. Following this incubation, larval survival was measured during 2 hr of photoactivation and once again 22 hr later in ambient lighting (which is insufficient for photoactivation). Whiskers indicate the 95% confidence interval (CI), and n indicates the number of mosquitoes. (PDF) [file pone.0310177.s002.pdf]

Yeast encapsulation of photosensitive insecticides increases toxicity against mosquito larvae while protecting microorganisms

Cole J. Meier, Veronica R. Wrobleski, and Julián F. Hillyer*
Department of Biological Sciences, Vanderbilt University, Nashville, TN, USA
Julian.hillyer@vanderbilt.edu

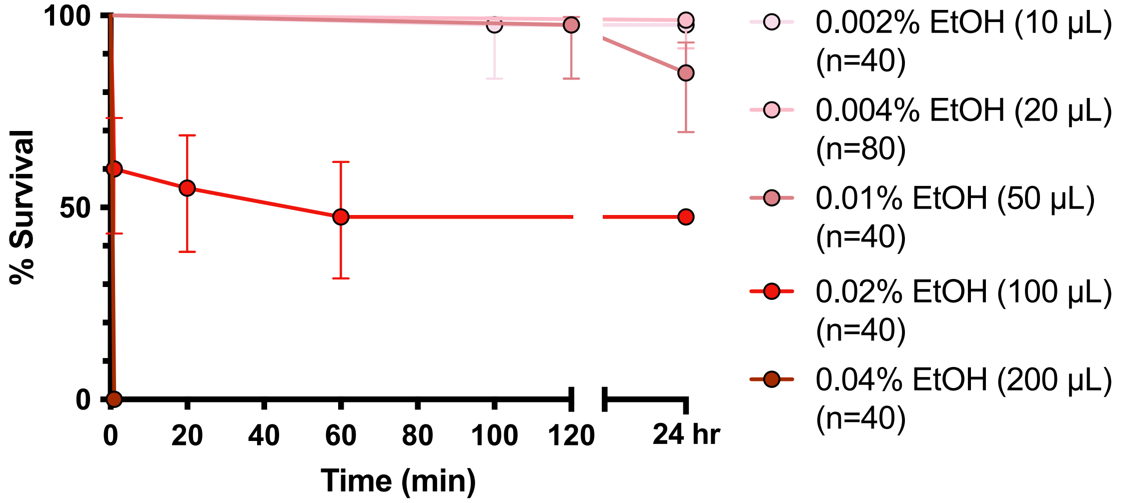

S1 Fig. Survival of larvae following exposure to ethanol. Larvae were incubated in the dark for 2 hr in water and either 0.002% (10 µL), 0.004% (20 µL), 0.01% (50 µL), 0.02% (100 µL), or 0.04% (200 µL) ethanol, for a total volume of 5 mL. Following this incubation, larval survival was measured during 2 hr of photoactivation and once again 22 hr later in ambient lighting (which is insufficient for photoactivation). Whiskers indicate the 95% confidence interval (CI), and n indicates the number of mosquitoes.
