# Supplementary material for: Yeast encapsulation of photosensitive insecticides increases toxicity against mosquito larvae while protecting microorganisms
Source: PLoS One. 2024 Oct 29;19(10):e0310177. doi: 10.1371/journal.pone.0310177 (PMC11521277; doi:10.1371/journal.pone.0310177)
Supplement: S2 Fig — Larval survival was measured after incubation with either 3 μM curcumin (A), 9 μM curcumin (B), 0.5 μM methylene blue (C), or 1 μM methylene blue (D) that was either non-encapsulated or encapsulated in yeast. Larvae were exposed for 2 hr in continued darkness, followed by an additional 2 hr of darkness and 22 hr of ambient lighting (insufficient for photoactivation). Time zero corresponds the initiation of the second 2 hr darkness incubation. Whiskers indicate the 95% confidence interval (CI), and n indicates the number of mosquitoes. (PDF) [file pone.0310177.s003.pdf]

Yeast encapsulation of photosensitive insecticides increases toxicity against mosquito larvae while protecting microorganisms

Cole J. Meier, Veronica R. Wrobleski, and Julián F. Hillyer*
Department of Biological Sciences, Vanderbilt University, Nashville, TN, USA
Julian.hillyer@vanderbilt.edu

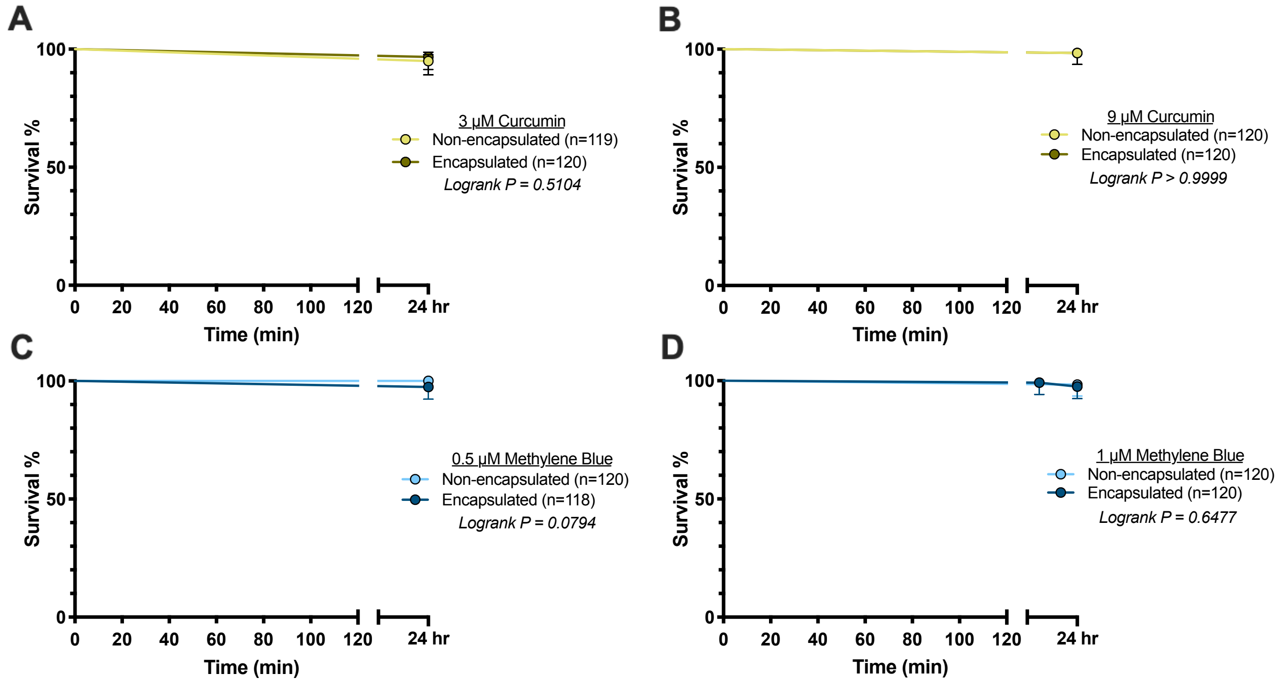

S2 Fig. Survival of larvae following exposure to non-encapsulated or yeast-encapsulated curcumin or methylene blue in the dark. Larval survival was measured after incubation with either 3 µM curcumin (A), 9 µM curcumin (B), 0.5 µM methylene blue (C), or 1 µM methylene blue (D) that was either non-encapsulated or encapsulated in yeast. Larvae were exposed for 2 hr in continued darkness, followed by an additional 2 hr of darkness and 22 hr of ambient lighting (insufficient for photoactivation). Time zero corresponds the initiation of the second 2 hr darkness incubation. Whiskers indicate the 95% confidence interval (CI), and n indicates the number of mosquitoes.
