# Supplementary material for: Yeast encapsulation of photosensitive insecticides increases toxicity against mosquito larvae while protecting microorganisms
Source: PLoS One. 2024 Oct 29;19(10):e0310177. doi: 10.1371/journal.pone.0310177 (PMC11521277; doi:10.1371/journal.pone.0310177)
Supplement: S3 Fig — Larval survival was measured following incubation with 50 μL, 200 μL or 500 μL of supernatant from the final wash of the curcumin (A) and methylene blue (B) yeast encapsulate that was added to 5 mL of water. Larvae were exposed for 2 hr in continued darkness, followed by 2 hr of photoactivation and 22 hr of ambient lighting (insufficient for photoactivation). Time zero corresponds to the initiation of the photoperiod. Data were analyzed using the Logrank Mantel Cox Test. Whiskers indicate the 95% confidence interval (CI) and n indicates the number of mosquitoes. (PDF) [file pone.0310177.s004.pdf]

Yeast encapsulation of photosensitive insecticides increases toxicity against mosquito larvae while protecting microorganisms

Cole J. Meier, Veronica R. Wrobleski, and Julián F. Hillyer*
Department of Biological Sciences, Vanderbilt University, Nashville, TN, USA
Julian.hillyer@vanderbilt.edu

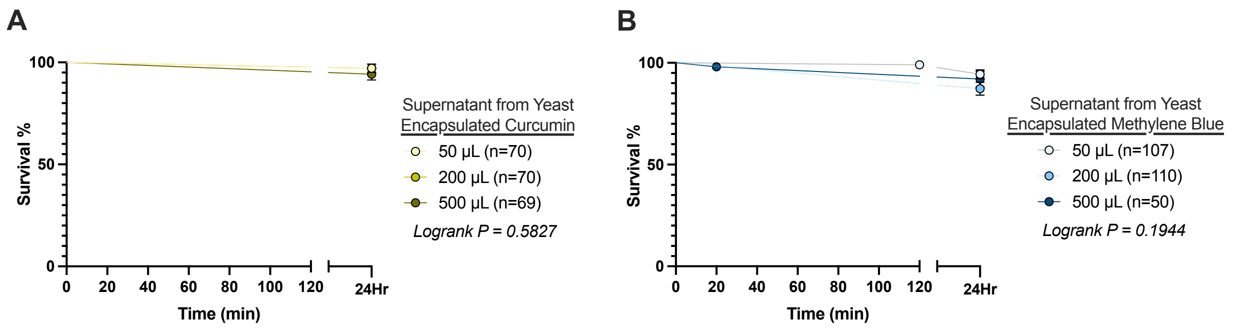

S3 Fig. Survival of larvae following exposure to the residual non-encapsulated curcumin and methylene blue remaining in the yeast encapsulate supernatant. Larval survival was measured following incubation with 50 µL, 200 µL or 500 µL of supernatant from the final wash of the curcumin (A) and methylene blue (B) yeast encapsulate that was added to 5 mL of water. Larvae were exposed for 2 hr in continued darkness, followed by 2 hr of photoactivation and 22 hr of ambient lighting (insufficient for photoactivation). Time zero corresponds the initiation of the photoperiod. Data were analyzed using the Logrank Mantel Cox Test (ns, $P > 0.05$). Whiskers indicate the 95% confidence interval (CI) and n indicates the number of mosquitoes.
