# Supplementary material for: Yeast encapsulation of photosensitive insecticides increases toxicity against mosquito larvae while protecting microorganisms
Source: PLoS One. 2024 Oct 29;19(10):e0310177. doi: 10.1371/journal.pone.0310177 (PMC11521277; doi:10.1371/journal.pone.0310177)
Supplement: S4 Fig — E. coli was either kept in the dark or exposed to a 30 min (A) or 2 hr (B) photoperiod before being plated and grown overnight. Colony forming units (CFUs) were counted to determine bacterial growth and survival. Column heights mark the mean, whiskers denote the S.E.M, and circles are the individual samples. Data for the 30 min Dark treatment is the same data reported as “No PSI” in S5 Fig; the experiments were conducted concurrently. (PDF) [file pone.0310177.s005.pdf]

Yeast encapsulation of photosensitive insecticides increases toxicity against mosquito larvae while protecting microorganisms

Cole J. Meier, Veronica R. Wrobleski, and Julián F. Hillyer*
Department of Biological Sciences, Vanderbilt University, Nashville, TN, USA
Julian.hillyer@vanderbilt.edu

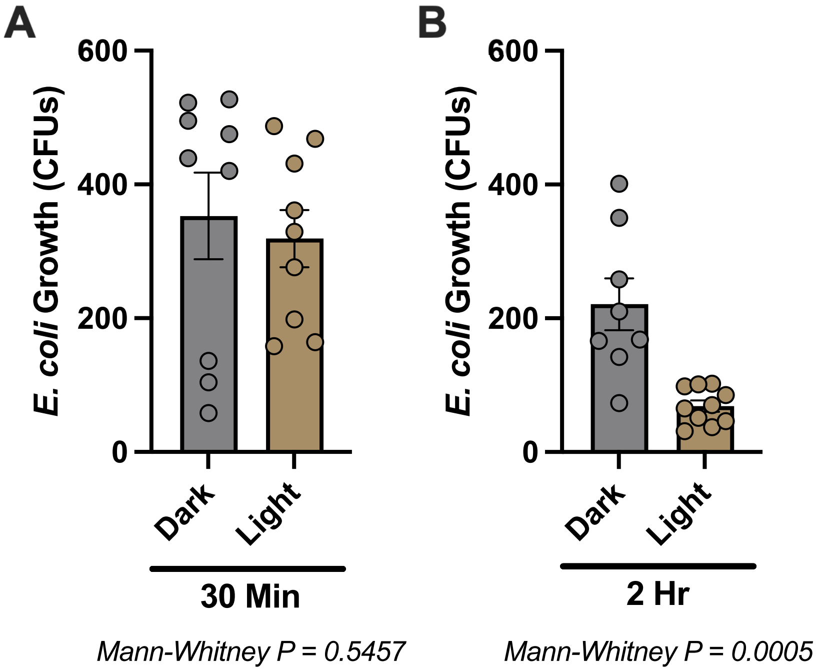

S4 Fig. Survival of *E. coli* following exposure to a 30 min or 2 hr photoperiod. *E. coli* was either kept in the dark or exposed to a 30 min (A) or 2 hr (B) photoperiod before being plated and grown overnight. Colony forming units (CFUs) were counted to determine bacterial growth and survival. Column heights mark the mean, whiskers denote the S.E.M, and circles are the individual samples. Data for the 30 min Dark treatment is the same data reported as "No PSI" in Supplemental Fig. S5; the experiments were conducted concurrently.
