# Supplementary material for: Yeast encapsulation of photosensitive insecticides increases toxicity against mosquito larvae while protecting microorganisms
Source: PLoS One. 2024 Oct 29;19(10):e0310177. doi: 10.1371/journal.pone.0310177 (PMC11521277; doi:10.1371/journal.pone.0310177)
Supplement: S5 Fig — E. coli were incubated with no PSI, 9 μM yeast-encapsulated curcumin, or 1 μM yeast-encapsulated methylene blue in the dark for 30 min, before being plated and grown overnight. Colony forming units (CFUs) were counted to determine bacterial growth and survival. Column heights mark the mean, whiskers denote the S.E.M, and squares are the individual samples. Data for the No PSI treatment is the same data reported as 30 min “Dark” in S4A Fig; the experiments were conducted concurrently. (PDF) [file pone.0310177.s006.pdf]

Yeast encapsulation of photosensitive insecticides increases toxicity against mosquito larvae while protecting microorganisms

Cole J. Meier, Veronica R. Wrobleski, and Julián F. Hillyer*
Department of Biological Sciences, Vanderbilt University, Nashville, TN, USA
Julian.hillyer@vanderbilt.edu

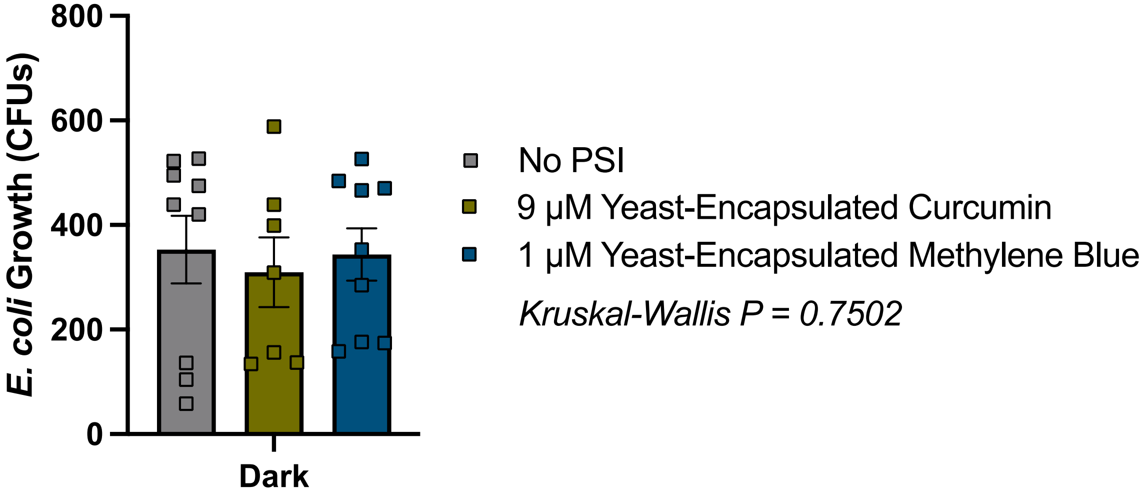

S5 Fig. Survival of *E. coli* following exposure to yeast-encapsulated curcumin and methylene blue in the dark. *E. coli* were incubated with no PSI, 9 µM yeast-encapsulated curcumin, or 1 µM yeast-encapsulated methylene blue in the dark for 30 min, before being plated and grown overnight. Colony forming units (CFUs) were counted to determine bacterial growth and survival. Column heights mark the mean, whiskers denote the S.E.M, and squares are the individual samples. Data for the No PSI treatment is the same data reported as 30 min "Dark" in Supplemental Fig. S4A; the experiments were conducted concurrently.
